# Heterogeneity Analysis of Esophageal Squamous Cell Carcinoma in Cell Lines, Tumor Tissues and Patient-Derived Xenografts

**DOI:** 10.7150/jca.52286

**Published:** 2021-05-10

**Authors:** Fayang Ma, Kyle Laster, Wenna Nie, Fangfang Liu, Dong Joon Kim, Mee-Hyun Lee, Ruihua Bai, Rendong Yang, Kangdong Liu, Zigang Dong

**Affiliations:** 1 Department of Pathophysiology, School of Basic Medical Sciences, College of Medicine, Zhengzhou University, Zhengzhou, China.; 2 China-US (Henan) Hormel Cancer Institute, Zhengzhou, Henan, 450008, China.; 3 College of Korean Medicine, Dongshin University, Naju-si, Jeollanam-do, 58245, Republic of Korea.; 4 Department of Pathology, Henan Cancer Hospital, The Affiliated Cancer Hospital of Zhengzhou University, Zhengzhou, Henan, 450008, China.; 5 The Hormel Institute, University of Minnesota, Austin, MN, 55912, USA.

**Keywords:** Esophageal Squamous Cell Carcinoma, Genetic Heterogeneity, Proteomics, Transcriptome, Heterograft, Bioinformatics.

## Abstract

Esophageal Squamous Cell Carcinoma (ESCC) is the predominant type of Esophageal Cancer (EC), accounting for nearly 88% of EC incidents worldwide. Importantly, it is also a life-threatening cancer for patients diagnosed in advanced stages, with only a 20% 5-year survival rate due to a limited number of actionable targets and therapeutic options. Increasing evidence has shown that inter-tumor and intra-tumor heterogeneity are widely distributed across ESCC tumor tissues. In our work, multi-omics data from ESCC cell lines, tumor tissue, normal tissue and Patient-Derived Xenograft (PDX) tissues were analyzed to investigate the heterogeneity among ESCC samples at the DNA, RNA, and protein level. We identified enrichment of ECM-receptor interaction and Focal adhesion pathways from the subset of protein-coding genes with non-silent mutations in ESCC patients. We also found that *TP53*, *TTN*, *KMT2D*, *CSMD3*, *DNAH5*,* MUC16* and* DST* are the most frequently mutated genes in ESCC patient samples. Out of the identified genes, *TP53* is the most frequently mutated, with 84 distinct non-silent mutation variants. We observed that p.R248Q, p.R175G/H, and p.R273C/H are the most common *TP53* mutation variants. The diversity of *TP53* mutations reveal its importance in ESCC progression and may also provide promising targets for precision therapeutics. Additionally, we identified the Olfactory transduction as the top signaling pathway, enriched from genes uniquely expressed in The Cancer Genome Atlas (TCGA)-ESCC patient tumor tissues, which may provide implications for the exact roles of the corresponding genes in ESCC. Cyclic nucleotide-gated channel subunit beta 1(CNGB1), a gene belonging to the Olfactory transduction pathway, was found exclusively overexpressed in ESCC. Expression of CNGB1 could serve as a marker, indicating potential diagnostic or therapeutic value. Finally, we investigated heterogeneity in the context of the ESCC PDX model, which is an emerging tool used to predict drug response and recapitulate tumor behavior *in vivo*. We observed trans-species heterogeneity in as high as 75% of the identified proteins, indicating that the ambiguity of proteins should be addressed by specific strategies to avoid drawing false conclusions. The identification and characterization of gene mutation and expression heterogeneity across different ESCC datasets, including various novel *TP53* mutations, ECM-receptor interaction, Focal adhesion, and Olfactory transduction pathways (*CNGB1*), provide researchers with evidence and implications for accurate research and precision therapeutic development.

## 1. Introduction

Esophageal cancer (EC) is a malignant cancer with a 15-25% 5-year survival rate worldwide, which is also the sixth leading cause of death from cancer and the eighth most common cancer 1, 2. EC has two main subtypes: Esophageal Squamous Cell Carcinoma (ESCC) and Esophageal Adenocarcinoma (EAC) 3. EAC has a glandular structure and arises primarily from Barrett's mucosa in the lower esophageal tube 4. In contrast, ESCC arises from stratified squamous epithelium and is more frequent in the proximal to middle esophagus 5. Annually, ESCC accounts for around 88% of the 450,000 EC incidents worldwide 6. Common treatment strategies for ESCC include esophagectomy, radiation therapy, chemotherapy, targeted therapy, and immunotherapy 4, 7. For ESCC patients in stage I (T1N0M0), esophagectomy is recommended as the standard treatment, and the 5-year survival rates could exceed 70% 8. Patients generally experience difficulties in swallowing, weight loss, and hoarseness prior to being diagnosed with advanced-stage ESCC. The outcomes of the current established therapeutics such as chemoradiotherapy for advanced stages or adjuvant chemoradiotherapy with surgery are very disappointing.

In recent years, targeted therapies have emerged as optional treatments for ESCC patients in advanced stages. Humanized monoclonal antibodies, such as trastuzumab which targets *HER2,* may be recommended for cases unsuitable for surgery with high *HER2* levels. Ramucirumab, a monoclonal antibody which targets *VEGF,* is also used in the combination with chemotherapeutic compounds such as paclitaxel. These modern immunotherapies could improve survival but the outcomes remain unsatisfactory 4.

Heterogeneity, from which tumor cells exhibit distinct phenotypes, is a complex molecular feature of ESCC that contributes to metastasis, tumor evolution and therapeutic outcomes 9. Recent Whole Genome Sequencing (WGS) and ChIP-seq datasets derived from ESCC samples have indicated inter- and intra-tumor heterogeneity in terms of histone acetylation, methylation, and various types of genetic alterations, which facilitate widespread transcriptional misregulation 9. The most frequently mutated genes reported in ESCC are *TP53*, *MLL2*, *NFE2L2*, *ZNF750*, *TGFBR2* and *NOTCH1*; while frequently upregulated signaling pathways include syndecan, Wnt and p63 related 10. Genomic sequencing of cancers, such as Non-Small-Cell Lung Cancer and melanoma, have yielded actionable targets such as EGFR-L858R and BRAF-V600E, respectively 11, 12. However, despite the extensive body of data derived from sequencing ESCC tumor tissues, results have not provided clinicians with putative actionable targets. Thus, innovative drug development strategies or breakthroughs in genome editing technology are urgently needed to translate basic research findings to clinically relevant results.

ESCC cell lines are one of the most common and accessible research materials. The ESCC PDX model also has specific advantages in therapeutic evaluation and mechanism studies. In the present work, gene mutation and expression data from ESCC cell lines and patient tumor tissues, proteome data from PDX tissues, and transcriptome data of normal esophageal tissue were integratively analyzed. The results illustrated that heterogeneity is widely dispersed among and between ESCC patient tumor tissues and established ESCC cell lines. In the context of gene mutations, we observed various *TP53* mutations in ESCC patient tumor tissues. Additionally, we identified Olfactory transduction (CNGB1) as a novel enriched signaling pathway from the subset of genes exclusively expressed in TCGA-ESCC patient tumor tissues. These findings provide novel evidence for further ESCC study, and highlight promising targets for the development of precision therapeutics.

## 2. Materials and Methods

### 2.1. Dataset collection, data processing and homogenization

12 Esophageal Squamous Cell Carcinoma (ESCC) cell lines were analyzed in this study: kyse30, kyse50, kyse70, kyse140, kyse150, kyse180, kyse220, kyse270, kyse410, kyse450, kyse510 and kyse520. The datasets detailing genetic mutation profiles for the 12 cell line were downloaded from the Cancer Cell Line Encyclopedia (CCLE, https://portals.broadinstitute.org/ccle) (Supplemental Data). The Ensembl Transcript IDs (ENSTs) of genes containing silent and non-silent mutations for each cell line were converted into Ensembl Gene IDs (ENSGs) and GeneNames (GN) using Ensembl BioMart (http://asia.ensembl.org/biomart; Database: Ensembl Genes 101, Dataset: Human Genes GRCh38.p13). Replicate IDs were removed automatically (Supplemental Data).

Prior to downloading the dataset detailing the genetic mutations in ESCC patients, Case ID's differentiating squamous cell carcinoma from adenocarcinoma were downloaded from the TCGA database (https://portal.gdc.cancer.gov/repository; Primary Site Filter: esophagus, Diagnoses Morphology Filter: 8070/3, 8071/3, and 8083/3 are classified as Squamous Cell Carcinoma); patient cases (N=80) fitting this criteria were chosen for inclusion. Initial diagnoses of all patients participating in the study were made between 2001-2013; patient samples were processed for sequencing between 2011-2014. We obtained mutation annotated files for the TCGA-ESCA cohort from Firebrowse (http://firebrowse.org; Cohort: Esophageal Carcinoma, Files: Mutation_Packager_Calls) and filtered the file names using the aforementioned patient Case IDs to obtain the subset of files detailing somatic mutations in ESCC patients. Relevant information including the name of the mutated gene, its genomic location, variant classification, and variant type are listed in the header of each file.

The transcriptome dataset of 10 ESCC cell lines (Supplemental Data) were downloaded from CCLE (https://portals.broadinstitute.org/ccle/data; expression unit: Transcripts Per Million). The transcriptome expression data of the TCGA ESCA cohort was downloaded from Firebrowse (http://firebrowse.org; Cohort: Esophageal Carcinoma, Files: illuminahiseq_rnaseqv2-RSEM_genes). ESCC-specific patient transcriptome data were obtained by cross-referencing header information with the aforementioned Case IDs obtained from the TCGA database. Transcripts Per Million (TPM) units were calculated for the ESCC-specific patient transcriptome data by multiplying the entries within the scaled_estimate column by 10^6^. Finally, we downloaded the transcriptome of normal human esophagus from the Human Protein Atlas (https://www.proteinatlas.org/). Transcripts in all datasets with TPM values ≥ 0.1 were flagged as detectable; transcripts not meeting these criteria were considered undetectable.

As the present analyses focused primarily on protein-coding genes, we downloaded a list of all protein coding genes from the Human Protein Atlas (19588 genes in Ensembl Gene [ENSG] ID) (Supplemental Data) to filter the datasets. Finally, the Ensembl Gene IDs of the ESCC cell line and ESCC patient transcriptome datasets were queried against the Ensembl Gene IDs of all protein coding genes obtained from the Human Protein Atlas. Ensembl Gene IDs not included in the list of all protein coding genes were excluded from subsequent analyses. Header-labeled original and processed data can be found in the supplementary material. Differential gene expression analysis was performed using ESCA transcriptome RNA-seq read count data downloaded from from the TCGA database. The TCGA-ESCA cohort was filtered using downloaded Case IDs to obtain ESCC transcriptome and normal tissue RNA-seq read count data. Transcript counts with less than 10 reads were identified and flagged; transcripts were excluded if more than 75% of their respective samples were flagged. Read counts detailing the transcriptome of normal and ESCC patient tissues were processed with the DESeq2 program in R using a standard pipeline 13. Entries with adjusted p-values less than 0.005, and exhibiting a log_2_(fold-change) > 1 or log_2_(fold-change) < -1 between normal and ESCC samples were identified for subsequent analyses.

CNGB1 expression data in 11 normal esophageal patient tissues was filtered from the Firebrowse ESCC transcriptome data. The diagrams comparing CNGB1 expression in different tumor tissues with corresponding normal tissues were downloaded from GEPIA 14, an interactive web server for analyzing RNA sequencing data derived from normal and tumor tissues, using a standard analysis pipeline.

### 2.2. Matching dissimilarity and correlation analysis

The bivariate correlation analysis for the number of silent mutations and the number of non-silent mutations was performed using the Pearson correlation coefficient. The number of silent mutations and non-silent mutations in each sample was graphed using a scatter plot against the horizontal and vertical axis in a Scatter Plot diagram. The linear regression equation (y = ax + b), R and R^2^ value were also calculated.

Mutation heterogeneity was calculated using a matching dissimilarity function. The union of all protein-coding genes containing non-silent mutations were expressed as Boolean vectors for each sample; each gene was assigned a fixed position along the vector and was denoted 0 (False) or 1 (True) based upon its mutation status in its respective sample. The matching dissimilarity is defined as 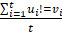, where u and v correspond to the Boolean vectors detailing the non-silent mapped mutations of each vector at position i. The matching dissimilarity output ranges bounded [0,1], with an output of 0 indicating no difference between input vectors while an output of 1 indicates maximum dissimilarity between input vectors. Calculation of expression heterogeneity was calculated using correlation distance. The correlation distance between u_i_ and v_i_ is defined as 1 - (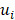-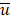)*(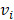-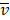)/(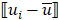
*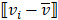), where u and v are vectors detailing the exome (in TPM) of samples. Matching dissimilarity and correlation distance analyses were computed using Mathematica Version 12.

### 2.3. The proteomic data derived from tissues of PDX-ESCC patients

The Patient-derived xenograft (PDX) model is established by subcutaneously implanting patient-derived tumor tissue into severe combined immunodeficiency (SCID) mice. PDX mouse models have been established and maintained for research in-house 15. NOD.CB17-*Prkdc^scid^*/J (NOD, Non-obese diabetic) mice were maintained in accordance with the guidelines of Laboratory Animal Welfare and Ethics Committee in Zhengzhou University. To guarantee the inter-tumor heterogeneity between different ESCC patients, two individual ESCC PDX models were processed for proteomic analysis. The iTraq Reagent 8-plex kit (#4381663, AB) (iTraq, Isobaric Tags for Relative and Absolute Quantitation) was used for quantitative proteomics processing based upon the manufacturer's instructions. In order to ensure the biological reproducibility and intra-tumor heterogeneity of the original patient tissue, four individual mice (tumor volume about 200 mm^3^) from each of the two ESCC PDX models were sacrificed and one piece of tumor tissue per mouse was resected. The resected tissues were cut and homogenized separately in 1.5 mL EP tubes using a tissue homogenizer. 100 mg of homogenized tissue from each tube was lysed in 0.8 mL RIPA (#R0010, Solarbio, containing 1mM PMSF, #P8340, Solarbio) (PMSF, Phenylmethylsulfonyl Fluoride; RIPA, Radioimmunoprecipitation) for 5 min, then centrifuged at 13,000g to extract the total proteins. The protein supernatants were transferred to new tubes and quantified using the BCA kit (#PC0200, Solarbio) (BCA, Bicinchoninic Acid). 200µg of total protein from each tube was transferred into new tubes and trypsinized, 100µg of fragmented peptides per tissue were processed to bond separately with eight isobaric tags (113-119, 121). The labeled peptides from the eight tissues were pooled, fractionated, and aliquoted into forty-eight tubes using high pH reversed-phase liquid chromatography (#1260 Infinity, Agilent Technologies). The fractions were symmetrically combined into 12 tubes of sample. Subsequently, 8 µL from each of these 12 samples was loaded and analyzed by tandem mass spectrometry (MS/MS) (ekspertTM Nano LC 415-AB SCIEX Triple TOF 5600). Fragmentation data from the resulting MS/MS spectrum was queried (under the cutoff of False Discovery Rate [FDR] < 0.01) against the UniProt database using the ProteinPilot software. A total of 5, 290 individual proteins were identified and used as the proteomic data for analysis. In calculating scores of the identified proteins, the matter of redundancy was considered and addressed. The assigned scores of identified proteins were treated as the total scores of all distinct peptides, and the distinct peptide sequences were identified as the single highest scoring fragments in the MS/MS spectrum. Peptide modifications and varying precursor charges along the identified peptide were considered irrelevant for subsequent analyses. Peptides more than eight residues in length were recorded in multiple different entries of proteins, with potential isoforms being grouped together. PDX data of human_origin (5290 proteins) and mouse_origin (4285 proteins) was obtained separately by querying the Uniprot database. The human Uniprot IDs were converted into ENSGs using the Ensembl database, which were used in the subsequent integrative analysis (Supplemental Data). The IDs used for trans-species heterogeneity analysis between human_origin and mouse_origin proteins were converted from ENSGs to GN format for use in analyses (Supplemental Data).

### 2.4. Open source tools used for bioinformatic analysis

Venn diagrams for overlapping regions of interest were created using the program listed at the vib-UGent Center for Plant Systems Biology webpage (http://bioinformatics.psb.ugent.be/webtools/Venn/). DAVID (The Database for Annotation, Visualization and Integrated Discovery) bioinformatics resources 6.8 (NIAID/NIH) was used for joint functional annotation and Kyoto Encyclopedia of Genes and Genomes (KEGG) signal pathways enrichment 16, 17. To avoid counting duplicated genes, the EASE Score, a modified Fisher Exact statistic, was calculated based on corresponding DAVID gene IDs. The EASE Score cutoff value was set to 0.1; scores exceeding this value were all considered redundant and excluded from subsequent analyses. The enriched KEGG pathways displayed were bounded by the thresholds, Max. Prob. ≤ 0.1 and Min. Count ≥ 2. The minimum gene count threshold for KEGG pathway analysis was 2, as pathways with one listed gene involved are unreliable.

The Cytoscape open source software platform and the String database were used for integrating and visualizing complex networks with attribute data 18, as well as for generating protein-protein interaction and association networks with increased coverage which support functional discovery in genome-wide experimental datasets, respectively 19. Homo Sapiens [9606] was selected as the organism for analysis. The list of 321 pathway terms corresponding with 7512 unique genes in KEGG_27.02.2019 was chosen as selected ontologies and the reference set for hypergeometric analysis. ClueGO was chosen as the functional analysis mode. PPI networks showed medium specificity between the extent of extreme global and detailed degrees, only pathways with p-Value ≤ .05 were shown; all unconnected nodes were hidden to increase figure legibility.

## 3. Results

### 3.1. Heterogeneity with respect to gene mutations and expression is prevalent across ESCC samples

**Figure 1:**
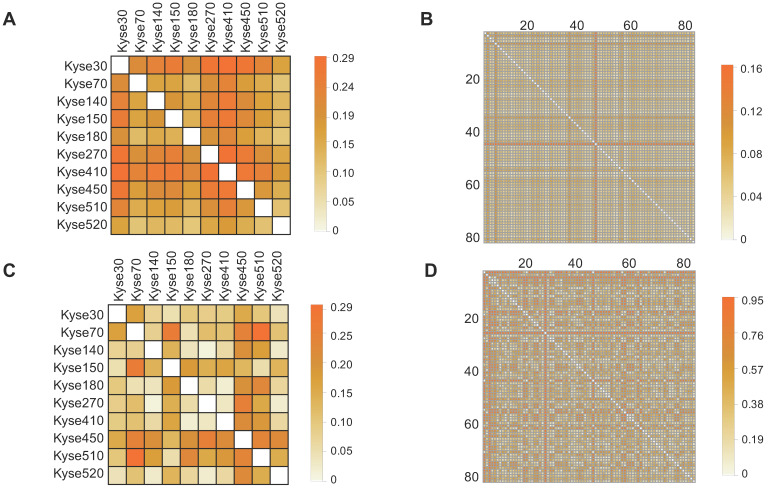
** Investigation of mutation and expression heterogeneity in CCLE-ESCC cell lines and TCGA-ESCC patient tissue.** (A and B) Heatmaps illustrating gene mutation heterogeneity as a function of matching dissimilarity in CCLE-ESCC cell lines (N=10) and TCGA-ESCC patient tissues (N=80), respectively (CCLE-ESCC : 4725 genes, TCGA-ESCC: 8510 genes). Darker hues indicate increased dissimilarity among ESCC samples. (C and D) Heatmaps illustrating expression heterogeneity as a function of transcriptome correlation distance in CCLE-ESCC cell lines (N=10) and TCGA-ESCC patient tissues (N=80), respectively (CCLE-ESCC: 15258 genes, TCGA-ESCC: 18335 genes).

To determine the prevalence of inter-sample heterogeneity at the genomic level across ESCC cell lines and patient tissues, we first determined the non-silent protein-coding gene mutations presented within each sample. Next, we constructed Boolean vectors representative of each sample's gene mutation profile and utilized a matching dissimilarity function highlight differences among the ESCC cell lines and ESCC patient tissues (see Materials and Methods 2.2). Our results showed that the matching dissimilarity of the mutation profiles within ESCC cell lines ranged between 0.19 to 0.29, with a mean of 0.24, while the dissimilarity between ESCC patients ranged between 0.02 and 0.16, with a mean of 0.05 (Fig. 1A, Fig. 1B). In the ESCC cell lines and patient tissues, the stark differences between the corresponding dissimilarities is largely correlated with two main factors, namely the length of the vector representing the mutation profile for each sample and the number of unique mutations in each patient sample. The given set of mutated genes identified in the patient tissues varies substantially, while there is a large overlap between the mutated gene profiles of the ESCC cell lines. This key difference leads to the creation of sparse vectors as inputs to the matching dissimilarity function that are assumed to be similar by virtue of the algorithm.

A more adequate metric to discern the degree of heterogeneity among ESCC cell lines and patient samples can be achieved by comparison of their respective transcriptomes. Utilizing this approach, the previously encountered caveat associated with Boolean vector encoding is circumvented, as each detectable gene expression is assigned a non-binary unit detailing its expression level. Thus, we compared transcriptome expression (in TPM units) heterogeneity across ESCC cell lines and patient tissue using the correlation distance function (see Materials and Methods 2.2). The results showed that correlation distance between the ESCC cell lines ranges between 0.02 and 0.30, with a mean of 0.10; within the TCGA patient tissues, the correlation distance ranges between 0.02 and 0.95, with a mean of 0.39 (Fig. 1C, Fig. 1D). The results suggest a tighter relationship between the expression profiles of the ESCC cell lines than between these of the TCGA patient tissues. One possible explanation for this is, within the cell lines, the larger subset of shared mutated genes that target the common transcription regulatory networks than in the tissue samples. Taken together, these observations illustrate variable degrees of heterogeneity among samples of ESCC cell lines and TCGA patient tissues. Importantly, these results highlight that the extensive variability of heterogeneity within TCGA tissues at the mutation and expression levels may not be adequately represented through use of cell lines.

### 3.2. The heterogeneity of non-silent mutations is widespread across ESCC samples

**Figure 2:**
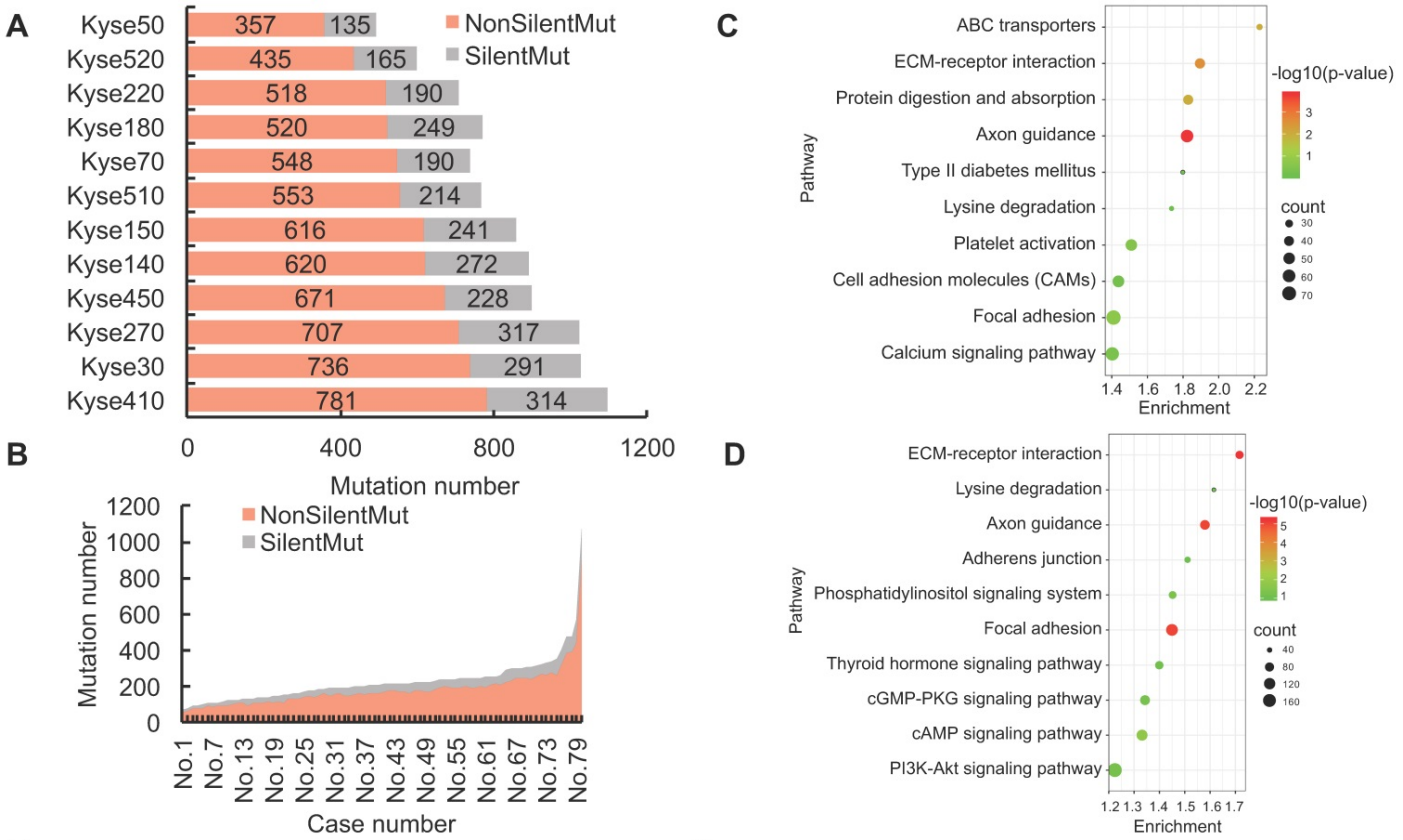

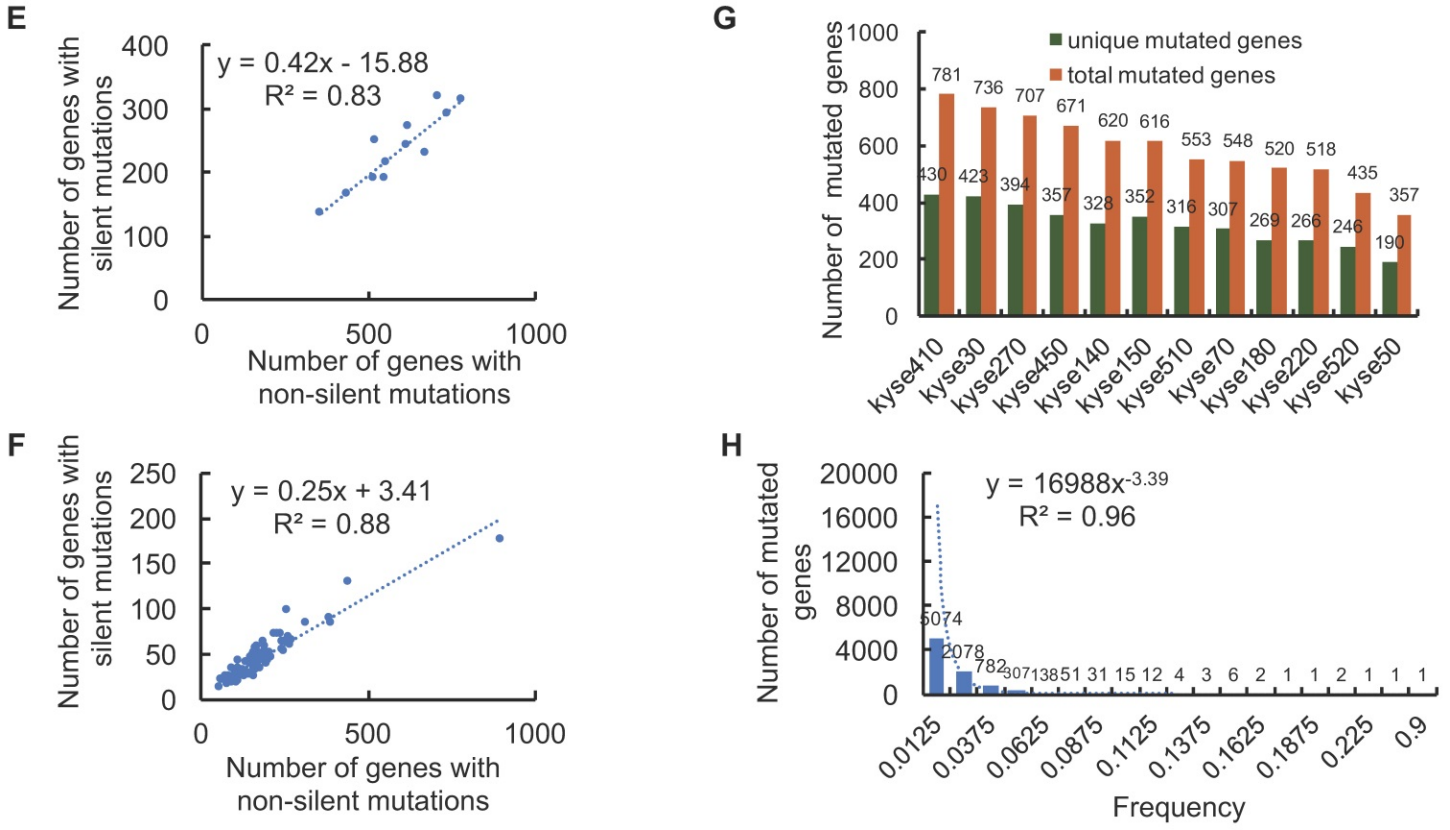
**The landscape of non-silent mutation heterogeneity in ESCC.** (A and B) The grey color indicates the number of genes with silent mutations, and the red color indicates genes with non-silent mutations across the ESCC cell lines (A) and patient tissues (B). (C and D) The top ten significantly enriched KEGG signaling pathways from genes harboring non-silent mutations in ESCC cell lines and patient tissues, respectively. (E and F) A positive correlation was observed between the number of genes with silent mutations and genes with non-silent mutations in ESCC cell lines and patient tissues, respectively. (G) The number of total mutated genes and unique mutated genes across the 12 ESCC cancer cell lines. (H) The frequencies of non-silent mutated genes across ESCC patient tumor tissues.

Genetic mutations could potentially result in the expression of dysfunctional proteins, and functionally impact their respective biological activities within the tumor cells. In order to reveal the landscape of genetic heterogeneity across different ESCC samples, we analyzed the non-silent mutations identified in ESCC cell lines and patient tissues. A total of 5190 non-silent mutations were observed in the 12 ESCC cell lines, with the ratio of non-silent mutations compared to total mutations ranging between 68-75% (Fig. 2A). The observed ratio range in the TCGA-ESCC patient tissues was between 73-87% (Fig. 2B). To determine which cellular pathways could be potentially influenced by genomic aberrations, we conducted KEGG enrichment analysis using the observed protein-coding genes harboring non-silent mutations. The top five most significantly enriched signaling pathways in ESCC cell lines were identified as Axon guidance, ECM-receptor interaction, Protein digestion and absorption, ABC transporters, and Focal adhesion pathways (Fig. 2C). By using the same pipeline of analysis in ESCC cell lines, the top five pathways identified in ESCC patient tissues were ECM-receptor interaction, Focal adhesion, Axon guidance, cAMP signaling pathway, and Phosphatidylinositol signaling system pathways (Fig. 2D). These signaling pathways are frequently dysregulated in a variety of cancers, which enhances confidence in the investigation of their respective mechanisms. In sum, this evidence indicates that the normal functions of the above signaling pathways could be extensively affected by the involved mutated genes and aid the precision therapeutic development.

We also found the number of genes with silent mutations and genes with non-silent mutations were positively correlated both in ESCC cell lines (R = 0.91, R^2^ = 0.83) and ESCC patients (R = 0.94, R^2^ = 0.88) (Fig. 2E, Fig. 2F), which indicated conservative correlations of silent mutations and non-silent mutations in ESCC samples. The unique non-silent mutations of each cell line showed a high level of genetic heterogeneity, which should be taken into consideration before being selected for precision oncology research. The numbers of unique non-silent mutated genes for each of the 12 ESCC cell lines were also analyzed. The ratio of unique non-silent mutated genes compared to total non-silent mutated genes in each ESCC cell line ranges between 51-57% (Fig. 2G). In total, 60% of the non-silent gene mutations were identified as unique to specific ESCC patients, which is slightly higher than 55% across the individual cell lines (Fig. 2G, Fig. 2H), and illustrates high-level heterogeneity with regards to mutational genotype across different ESCC samples.

### 3.3. A variety of novel non-silent mutations in *TP53* in ESCC samples

**Figure 3:**
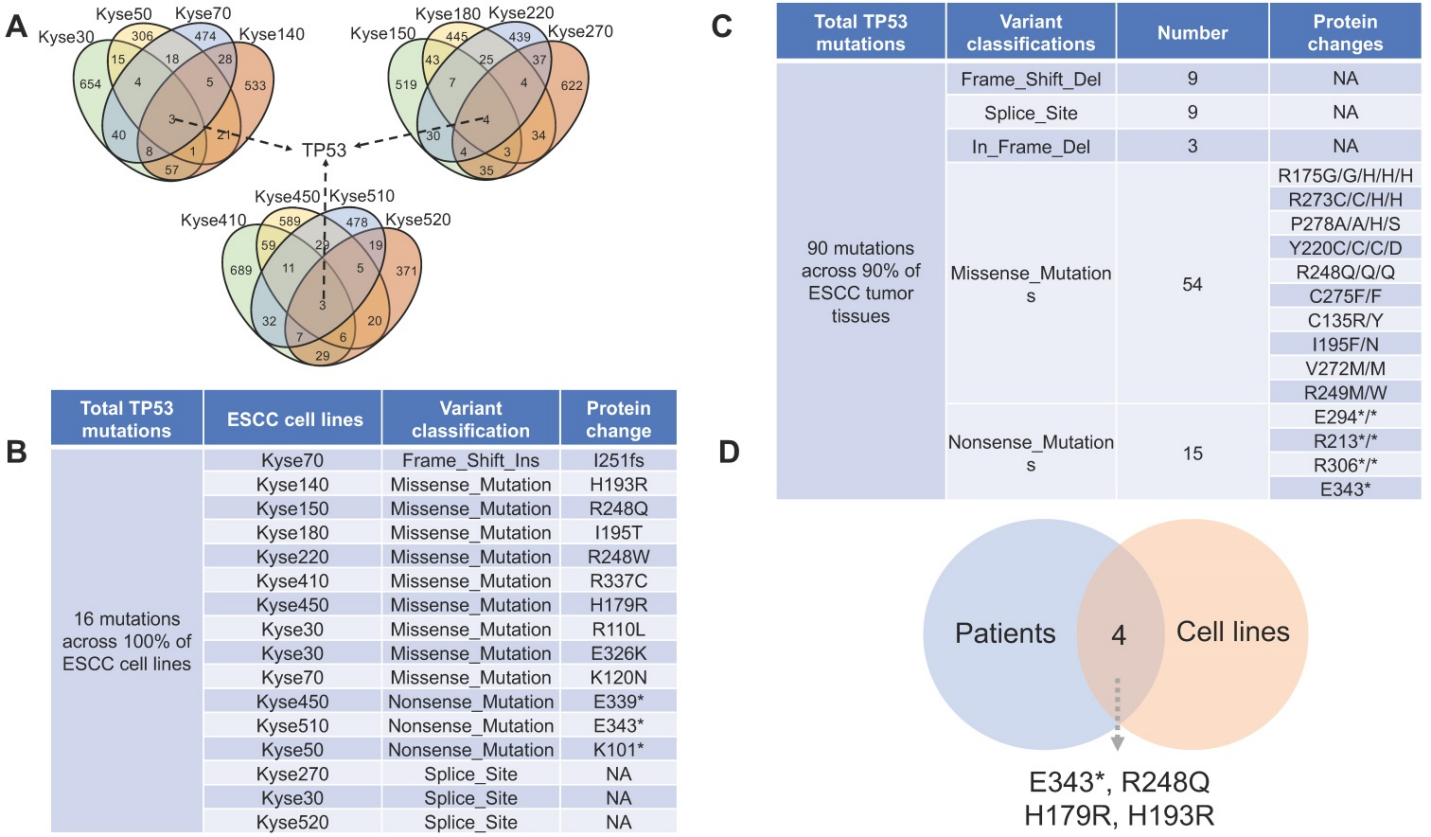
** The distribution of *TP53* non-silent mutations across ESCC samples.** (A) *TP53* is the only mutated gene shared by all 12 ESCC cell lines. (B) A total of 16 *TP53* non-silent mutations were identified across the 12 ESCC cell lines. On average, more than one *TP53* mutation per cell line was observed. (C) A total of 90 *TP53* non-silent mutations are distributed across 90% of ESCC patient tumor tissues (a complete list in the supplemental data). (D) Overlap diagram illustrating commonly shared TP53 non-silent mutations between ESCC patients and ESCC cell lines.

P53 is a well-known tumor suppressor that mediates cellular senescence and apoptosis, a variety of deleterious mutations have been identified in many types of cancers 20. Of all the identified mutated genes, genetic aberrations of *TP53* are prevalent in 100% of the 12 ESCC cell lines (Fig. 3A, Fig. 3B) and 90% in ESCC patient tissue (Fig. 3C). Thus, experimental design involving DNA damage response and apoptosis pathways in ESCC cell lines should be prudent, as canonical signaling pathways may be affected by dysfunctions of mutant p53 protein. In total, 84 distinct *TP53* non-silent mutations are categorized into five classes: Missense_Mutations, Nonsense_Mutations, Frame_Shift_Del, Splice_Site and In_Frame_Del. with p.R248Q, p.R175G/H, and p.R273C/H being identified as the most frequent mutation variants (Supplemental Data). Four *TP53* mutations (E343*, R248Q, H179R and H193R) are commonly shared by ESCC cell lines and patient tissues (Fig. 3D), which may make them feasible candidates for *in vivo* and *in vitro* precision research. Of the *TP53* mutations shared between ESCC cell lines and patient tissues, R248Q, H193R, and H179R have been previously reported to be associated with carcinogenesis in different types of cancer 21-23. However, the majority of other* TP53* mutations have not been reported as contributing to ESCC carcinogenesis. Thus, future research detailing the functional impact of deleterious *TP53* mutations, alongside with breakthroughs in drug development and genome editing technologies, would likely facilitate clinical translation of basic research.

### 3.4. Genomic aberrations are heterogeneously distributed across the un-transcribed and actively transcribed protein-coding genes in ESCC samples

**Figure 4:**
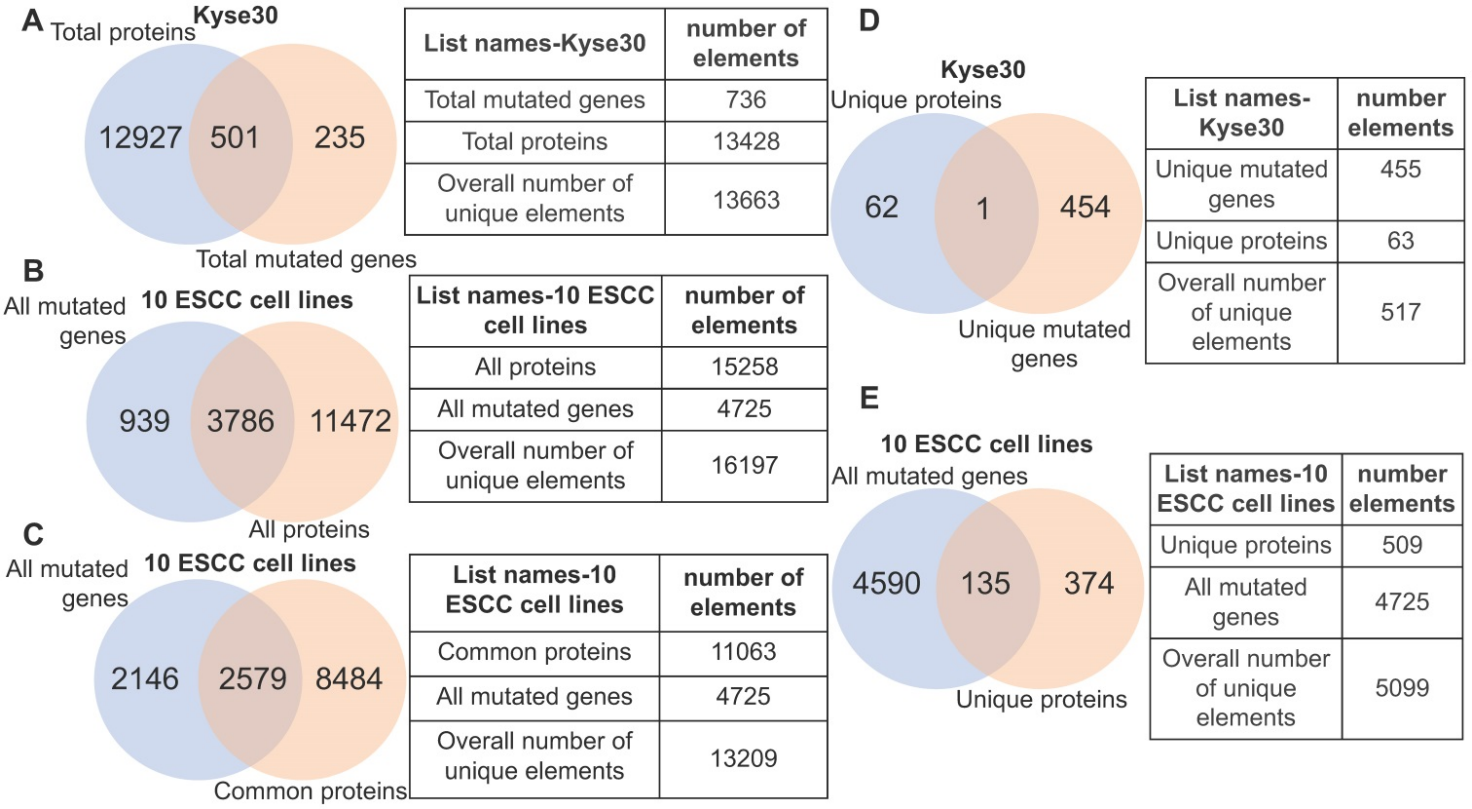
**Heterogeneity illustrated by integrative analysis of gene mutation and protein-coding transcriptome.** (A) 501 mutant proteins specific to kyse30 were revealed after overlapping total proteins with the total non-silent mutated genes in kyse30. (B) All mutated protein-coding genes were overlapped with all proteins expressed in the 10 ESCC cell lines. A subset of 3786 mutant proteins, a subset of 11472 proteins without mutations, and a subset of mutated but untranscribed genes were identified. (C) A subset of mutated genes commonly expressed in all the ten ESCC cell lines were observed. (D) Only one mutant protein was identified in the intersection of kyse30-specific proteins and kyse30-specific mutated genes. (E) All the unique proteins for each individual ESCC cell line were overlapped with all mutated genes, producing a subset of 135 mutant proteins unique across the 10 ESCC cell lines.

Although gene mutation may influence the normal functions of protein, the corresponding gene must be expressed. With this in mind, we analyzed the fraction of gene mutations that may not necessarily contribute to cancer phenotype due to transcriptional inactivity by querying the identified gene mutations of each ESCC cell lines against its actively transcribed protein-coding gene set. In the case of ESCC cell line kyse30, we observed that 3.7% (501/13428) of the protein-coding genes possessed non-silent mutations, while 235 protein-coding genes harboring non-silent mutations are un-transcribed (Fig. 4A). Within the ten ESCC cell lines we observed that 24.8% (3786/15258) of all transcribed protein-coding genes harbor non-silent mutations. Importantly, this indicates that the normal function of the corresponding translated proteins would be potentially affected (Fig. 4B). A subset of 939 mutated genes are un-transcribed within the analyzed ESCC cell lines, suggesting that they are likely passenger mutations that do not contribute to the cancer phenotype (Fig. 4B). Roughly 23% of commonly transcribed genes in cell lines are mutated, which provided a subset of promising targets (Fig. 4C). Interestingly, only one gene (*TULP2*) out of the 455 mutated genes specific to kyse30 is transcribed at a detectable level (Fig. 4D); this gene has not yet been associated with carcinogenesis. This finding is not specific in kyse30; unique un-transcribed mutations were observed in all other ESCC samples (data not shown). Altogether, 26.5% (135/509) of the total unique proteins harboring non-silent mutations are specific to certain individual ESCC cell lines (Fig. 4E). The above evidence illustrates that non-silent protein-coding mutations are extensively distributed across ESCC cell lines. Focus on the subset of commonly transcribed genes harboring non-silent mutations should facilitate therapeutics development generalized for ESCC patients.

### 3.5. A subset of proteins with bi-species homology illustrated trans-species heterogeneity in PDX model

**Figure 5:**
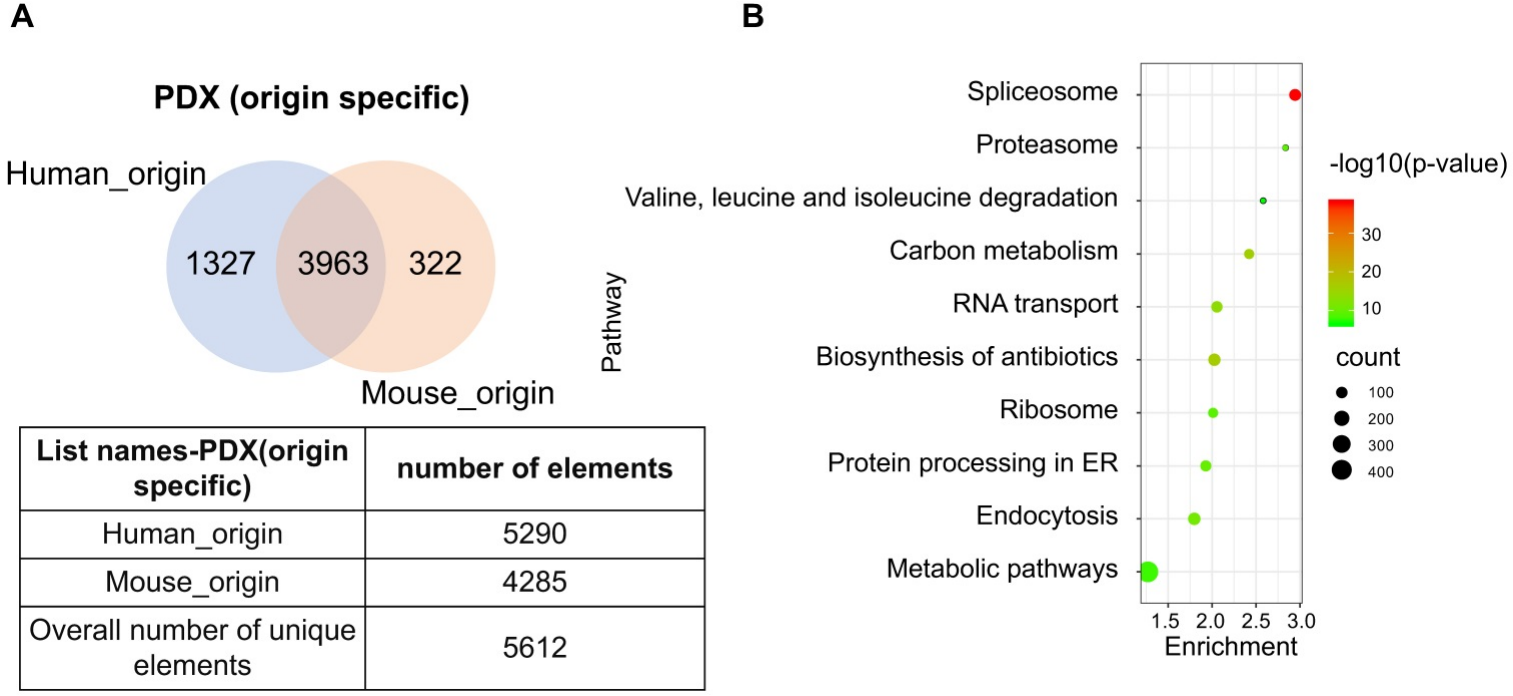
**A subset of proteins, termed Possibly of Murine Origin (PMO) proteins, was identified in the PDX model.** (A) The overlapping diagram demonstrated a subset of 3963 proteins was commonly shared by both origins, and the subsets of identified proteins with clear human_origin or mouse_origin in PDX tissue. (B) The top ten signaling pathways with the most significance were enriched from the subset of PMO proteins.

The PDX model is an emerging tool that provides prospects for personalized therapeutics. As the implanted tissues are directly resected from patient tumors, this model mimics the heterogeneity of cell types in human tumor tissue. However, implantation of human tissue into a murine host contributes to the chimeric nature of PDX, which produces a heterogeneous proteome with trans-species homology. Due to the commonly shared peptides between human and murine protein orthologues, the species of some proteins may not be accurately identified. To determine the extent of this phenomena, original data from two PDX-ESCC models were analyzed to determine proteins with human, murine, and ambiguous origins. Two proteomic datasets were identified independently by querying the same pool of peptide sequences against the human and mouse UniProt database, respectively. The list of human_origin proteins (N=5290) and the list of murine_origin proteins (N=4285) were overlapped, producing a subset of 3963 proteins shared by both species. The subset of proteins shared between species was termed as Possibly of Murine Origin (PMO) due to the indeterminate nature (Fig. 5A). Since cellular processes function via a variety of interconnected signaling pathways, misidentified signatures may provide spurious insights due to the indeterminate origin of proteins. Thus, it is necessary to clarify which signaling pathways these PMO proteins (3963 proteins) are enriched in. The results showed that the top five signaling pathways are Spliceosome, Biosynthesis of antibiotics, Carbon metabolism, RNA transport, and Endocytosis (Fig. 5B). The Spliceosome, RNA transport, and Endocytosis pathways have been reported being related with ESCC carcinogenesis 24-26. As *in vivo* models are the gold standard in cancer research, it is important to minimize the ambiguity of proteomic data derived from PDX, and the exact species of the PMO proteins should be thoroughly clarified.

### 3.6. The heterogeneity of gene expression across different ESCC datasets

**Figure 6:**
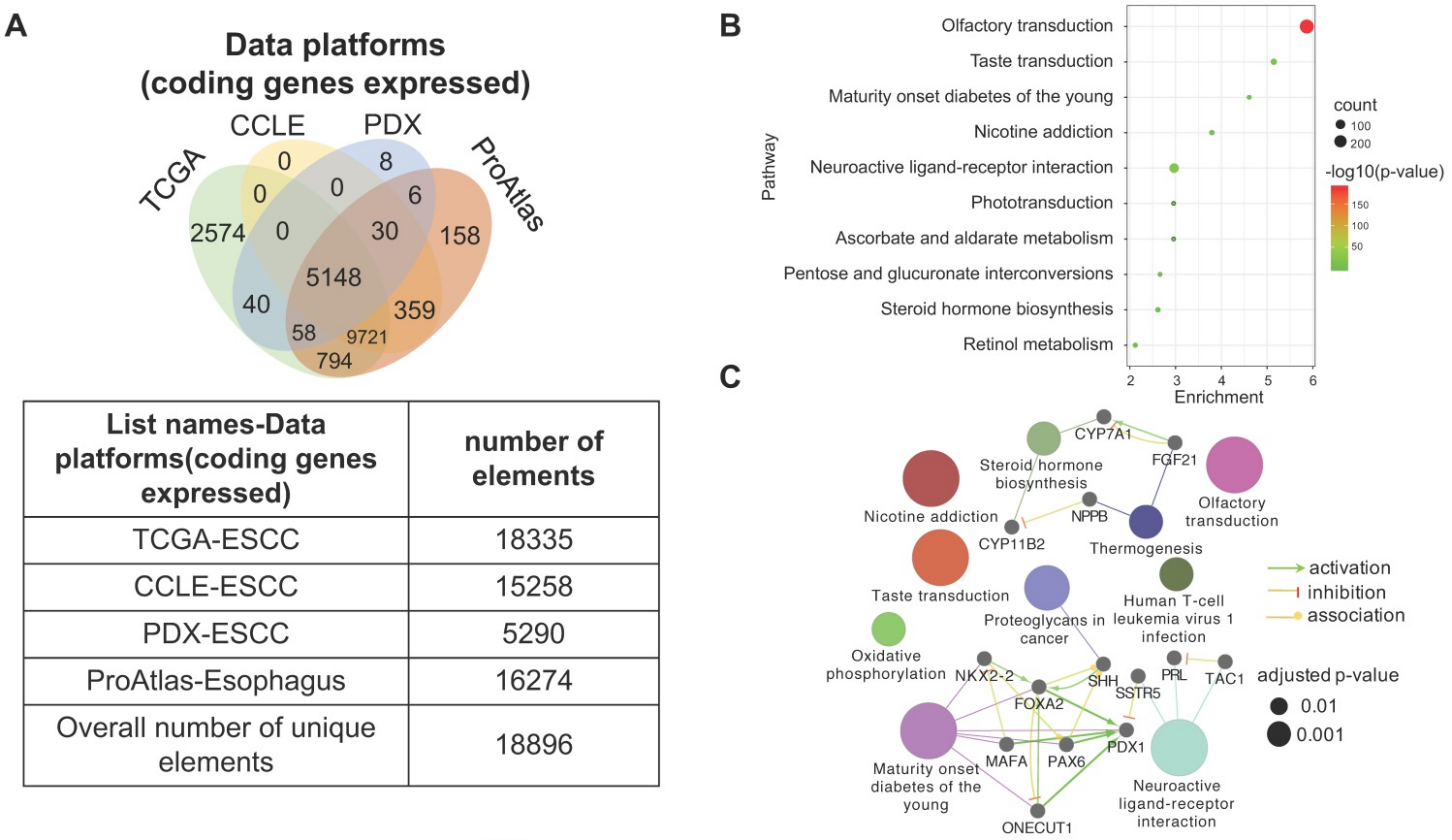

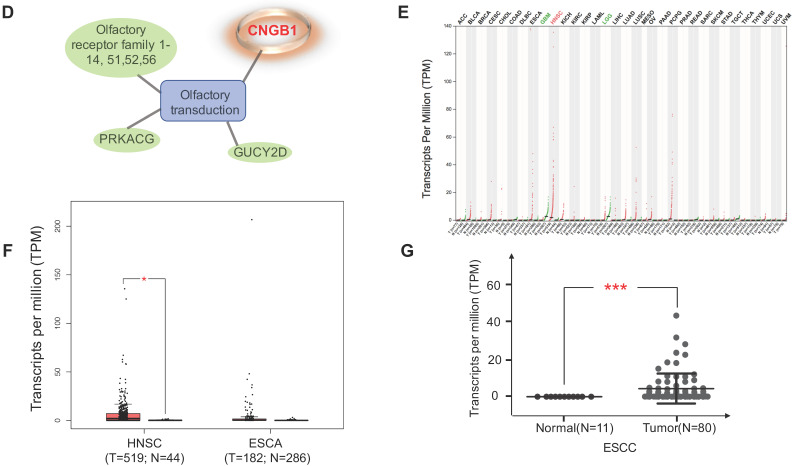
** A subset of 2574 uniquely expressed genes specific to TCGA-ESCC patient tumor tissues was identified.** (A) Diagram illustrating the unique and shared subsets of gene expression after overlapping the four datasets from TCGA-ESCC Tissues, CCLE-ESCC Cell Lines, Protein Atlas-Normal Esophagus Tissue and PDX-ESCC Tissues. (B) The top ten significant signaling pathways enriched from the 2574 TCGA-ESCC Unique genes. (C) The corresponding PPI and networks of signaling pathways derived from TCGA-ESCC Unique genes. (D) 293 genes residing within the Olfactory transduction pathway, out of which 290 genes belong to the Olfactory receptor family. (E) The expression landscape of CNGB1 across different Tumor-Normal tissue pairs. (F) The expression of CNGB1 in ESCA and HNSC. (G) The comparison of CNGB1 expression between 80 ESCC samples and 11 normal esophageal tissues. Significance was assessed using the Mann-Whitney test. * indicates *p<.05*, *** indicates *p<.001*.

Four datasets were integratively analyzed for better understanding the expression heterogeneity of protein-coding transcriptome in ESCC cell lines and patient tissues. The four datasets, detailing expression of 15258, 18335, 16274, and 5290 genes, were derived from CCLE-ESCC Cell Lines, TCGA-ESCC Tissues, Protein Atlas-Normal Esophagus Tissue, and PDX-ESCC Tissues, respectively. Subsets of genes unique to each dataset as well as gene subsets shared between datasets were produced via overlapping. The results illustrate that three out of the four datasets possess unique subsets of detectable genes, with as many as 2574 (14.04% of 18335) genes solely expressed in TCGA-ESCC Tissues (TCGA-ESCC Unique), 158 (9.71% of 16274) genes in Protein Atlas-Normal Esophagus Tissue (ProAtlas-Esophagus Unique), and 8 (0.15% of 5290) proteins in the PDX-ESCC Tissues (PDX-ESCC Unique) (Fig. 6A). We conducted KEGG pathway enrichment analysis and visualized protein-protein interaction (PPI) networks using TCGA-ESCC Unique, the largest of the identified subsets (Fig. 6B-C). Interestingly, the top three significantly enriched pathways correspond to Olfactory transduction, Neuroactive ligand-receptor interaction, and Taste transduction; all of which are related with sensory and neurological signal transmission. Nearly 99% (290/293) of the genes identified within the Olfactory transduction pathway belong to the olfactory receptor family. The remaining 3 identified genes were PRKACG, GUCY2D, and CNGB1 (Fig. 6D). Interestingly, after analyzing the CNGB1 expression landscape across different types of normal-tumor tissue pairs, we found that it was only significantly over-expressed in Head and Neck Squamous Carcinoma (HNSC) (Fig. 6E) . We observed that CNGB1 is not significantly over-expressed in ESCA (Fig. 6F); however, upon filtering the ESCA cohort for ESCC cases, we found that CNGB1 is significantly over-expressed in ESCC patient tissues (Fig. 6G). The Olfactory transduction and Neuroactive ligand-receptor interaction pathways were also observed to be significantly enriched in lung cancer and glioblastoma 27, 28, indicating potential diagnosis and therapeutic value.

## 4. Discussion

### Background of ESCC

ESCC is the most common type of EC, with a 15 to 20% 5-year survival rate worldwide, though in some countries the 5-year survival rate has improved by 15%. A key contributor to the high mortality rate of ESCC is late-stage diagnosis, as nearly 40% of incidences have metastasized by the time of diagnosis 29, 30. Histologically, EC is classified into two main subtypes, EAC and ESCC. Each subtype possesses distinct molecular characteristics. ESCC more closely resembles Head and Neck Squamous Carcinoma (HNSC) than EAC, and focal amplification of TP63, SOX2, and CCND1 are more pronounced 10. In contrast, focal amplifications of VEGFA, ERBB2, and GATA4/6 are more prevalent in EAC 10. EAC shares a similar molecular feature with gastric adenocarcinoma, and is prevalent in western countries and U.S. General risk factors for ESCC include cigarette smoking, alcohol consumption and radiation exposure; however, there may be complex interactions from the impact of different risk factors. Accumulated evidence has showed a great variance in geographical distribution of ESCC 31. For instance, several nations report high incidence rates of ESCC despite prohibition of alcohol consumption 32. An integrative analysis of 15 cohort studies concluded that supplementing the diet with folate could potentially reduce the risk of ESCC mortality, while alcohol consumption may increase the risk 33. Although studies have attempted to determine genetic, environmental, and dietary contributions to ESCC carcinogenesis in areas with high-rate ESCC incidences 34, 35, the exact risk factors and etiology have not been thoroughly elucidated.

### TP53 mutation heterogeneity in ESCC

Cancer is a heterogenous disease caused by genetic aberrations that contribute to uncontrolled cellular division, invasion, and metastasis. Additionally, copy number variations, structural aberrations, and altered DNA methylation also extensively contribute to tumor heterogeneity. Studies have also revealed ESCC heterogeneity at both the genomic and epigenetic level 36, 37. Around 40% oncogenic aberrations occurring in genes such as *KIT*, *PIK3CA*, *MTOR,* and *NFE2L2* were heterogeneously distributed in sub-clones of ESCC patient tumor tissues, while genetic alterations in *TP53*, *ZNF750,* and *MLL2* occur during earlier stages and are more predominant 38. Our analysis illustrated that non-silent protein-coding mutation profiles distributed across ESCC cell lines and patient tissues are heterogeneous and diverse, with *TP53*, *TTN*, *KMT2D*, *CSMD3*, *DNAH5*,* MUC16,* and* DST* being the most frequently mutated genes. These observations are supported by recent research 39, 40; however, with the exception of *TP53*, the exact role of these mutated genes in ESCC has not been clearly elucidated. Further research is needed to confirm whether these mutated genes contribute to ESCC carcinogenesis.

*TP53* is reported to be the most frequently mutated gene in ESCC 41; however, no shared mutated gene across samples has been identified. Indeed, 10% of TCGA-ESCC patients in our analysis are wild type for *TP53*. Mouse model studies have demonstrated that not all p53 mutations are functionally equivalent, and increasing evidence has shown that certain mutated *TP53* products gain additional properties that may result in equally deleterious consequences as functionally null mutants 20. In our analysis, the most frequent mutation variants of *TP53* are p.R248Q, p.R175G/H, p.R273C/H, and p.Y220C, which are functionally relevant missense mutations within the DNA-binding domain of p53. Evidence suggests that the p.R175H mutation could abrogate the tumor suppressive function of p53, simultaneously contributing to an oncogenic role for p53 42. Additionally, activation of the c-Met and STAT1 signaling axes, which facilitate ECM invasion, were correlated with expression of p53-R175H in ESCC cell lines 43-45. p53-R175H may be involved in the resistance of induced apoptosis 46, and has also been implicated in desensitizing ESCC tumors to Fas-mediated anchorage-independent death via a FAK-dependent mechanism 47. Aside from the reported p53-R175H in ESCC, p.R282W and p.R248W were reported in early stages of Barrett's adenocarcinoma 48. Our analysis revealed as many as 84 distinct *TP53* mutations. Aside from the p53-R175H mutation, the exact roles of the remaining 81 TP53 mutations in ESCC carcinogenesis have not been reported. A recent study has suggested that adoptive T-cell therapy could potentially be developed targeting mutated p53 p.R175H in multiple types of cancer 49. Precision therapeutics against the various subtypes of *TP53* mutations are proposed to be further investigated.

### Inter-sample and intra-sample heterogeneity in ESCC

Genomic sequencing has greatly improved the understanding of cancer heterogeneity 37. With respect to inter-tumor heterogeneity, three distinctive ESCC types were classified via the multi-omics analysis of 90 ESCC patients 10. However, the results and conclusions derived from basic research studies have not yet been translated to clinical practice and benefit ESCC patients. Signaling pathways enriched from mutant proteins in ESCC may provide insights into potentially actionable targets, which are urgently needed for the development of working therapeutics. Our analysis illustrated that the subset of mutated protein-coding genes are enriched in ECM-receptor interaction and Focal adhesion pathways in TCGA-ESCC patients. A subset of 614 genes was also enriched in the same pathways in previous a ESCC study 50. The expression level of these genes was negatively correlated with the expression level of miR-30b-5p; better prognosis was observed in ESCC patients with higher miR-30b-5p level 50. Evidence also showed that ECM-receptor interaction and Focal adhesion pathways were enriched from deregulated microRNA and mRNA in ESCC with respect to paired adjacent tissues 51. In sum, the above evidence suggests that a network of ECM-receptor interaction and Focal adhesion pathways enriched from mutant genes may play an important role in ESCC and provide potential actionable targets for precise therapeutics.

The integrative analysis of the four different datasets showed heterogeneous expression patterns with respect to commonly shared and unique gene subsets. The genes shared between ESCC patient tumor tissues and cell lines could be studied feasibly both *in vitro* and *in vivo*. However, there is some concern that these genes may harbor distinct mutations across different ESCC samples. As a result, protein function and oncogenic phenotypes could be potentially affected across samples 52. This is particularly relevant, for as high as 46.4% of all the expressed genes in ESCC patient tissues harbor non-silent mutations in our observation.

Tumor tissues are always analyzed as a single object by canonical sequencing techniques, the shortcoming of which is the possibility of overlooking worthwhile information. Our integrative analysis revealed a subset of 30 genes shared between the CCLE and PDX tumor tissues, but excluded in patient tissue (Fig. 6A). One study used a similar analysis pipeline and observed that CD44, which is a consensus marker of breast cancer, is only overexpressed in breast cancer PDX tissue and cell lines, but not in clinical samples 53. The explanation for this is that the subsets of genes is detectable in samples with high tumor cell content, but are likely occluded by substantial amounts of stroma in clinical tumor tissue.

Carcinogenesis and progression are modulated by a myriad of recruited cells including inflammatory cells, stromal cells, and vasculature that constitute the tumor micro-environment *in vivo* 54, 55. The observation of unique subsets of expressed genes in patient tumor tissue and PDX tumor tissue (Fig. 6A) are likely the result of interactions between the microenvironment and tumor cells. The Olfactory transduction (11%), Neuroactive ligand-receptor interaction signaling pathways (4%), and Taste transduction (1%) are the top three signaling pathways enriched from the unique subset of genes identified in patient tumor tissue. Interestingly, the Olfactory transduction and Neuroactive ligand-receptor interactions pathways were also found to be significantly enriched in lung cancer and glioblastoma 27, 28. CNGB1, a member in the Olfactory transduction pathway, was identified being significantly upregulated both in ESCC and HNSC, which is consistent with previous evidence supporting their similarities 10. CNGB1 is a subunit of the cyclic nucleotide-gated ion channels which specifically mediates sensory signal transduction in olfactory sensory neurons and retinal photoreceptors cells 56. The tumorigenic role of CNGB1 has not been reported in ESCC. Nonetheless, its overexpression in ESCC may correspond to the role of oncogene. Further research is needed to clarify its validity as a potential target or diagnostic marker. GRM3, a gene belonging to the Neuroactive ligand-receptor interaction pathway, was found to be up-regulated in esophageal tumor tissue using a cDNA microarray, while pathway member CCKAR was recommended as a biomarker for the early detection of ESCC 57, 58. Only one study has suggested an association between the Taste transduction pathway and ESCC risk 59. Few studies have reported the exact role of the aforementioned signaling pathways which may constitute potential targets for ESCC.

Intra-tumor heterogeneity has provided insight into ESCC tumorigenesis and progression 38. Multiple cell types assemble the tumor stroma and contribute to tumor development 54. The degree of intra-tumor heterogeneity in cancers such as HNSC, chronic lymphocytic leukemia, and hepatocellular carcinoma, is closely related with therapeutic responses and overall survival time. In the PDX model, stroma of murine origin is recruited and embedded within the patient tumor tissue, producing a complex mosaic comprised of both human_origin and mouse_origin cells. In our PDX proteomic analysis, as high as 75% of identified human proteins possessed indistinguishable homology with identified mouse proteins, which is termed as “trans-species” heterogeneity. This phenomenon illustrates that the ambiguous proteins should be thoroughly validated before coming conclusions. Strategies, attempting to solve the ambiguity of proteomes in PDX, have already been previously reported. gpGrouper is a peptide grouping algorithm for gene-centric inference and quantitation of bottom-up proteomics data in PDX, which precisely distinguishes tumor content without elimination of species-shared peptides 60. Incorporation of tools such as gpGrouper into proteomic analysis pipelines will confer additional confidence to research results; however, target confirmation using a panel of human patient tissues would provide the most conclusive result.

## Conclusion

In the present study, integrative analysis was performed using datasets derived from ESCC patient tissue, ESCC cell lines, and ESCC PDX models. Our results illustrated extensive heterogeneity at the genome and transcriptome level in ESCC cell lines and patient tissues. Additionally, we observed trans-species proteomic heterogeneity within PDX tumor tissues. The identification and characterization of gene mutation and expression heterogeneity, across different ESCC datasets, including various novel *TP53* mutations, ECM-receptor interaction, Focal adhesion, and Olfactory transduction pathways (*CNGB1*), provide researchers with evidence and implications for accurate research and precision therapeutic development.

## Supplementary Material

Supplementary figures and tables.
